# Animated Graphics for Comparing Two Risks: A Cautionary Tale

**DOI:** 10.2196/jmir.2030

**Published:** 2012-07-25

**Authors:** Brian J Zikmund-Fisher, Holly O Witteman, Andrea Fuhrel-Forbis, Nicole L Exe, Valerie C Kahn, Mark Dickson

**Affiliations:** ^1^Department of Health Behavior and Health EducationSchool of Public HealthUniversity of MichiganAnn Arbor, MIUnited States; ^2^Department of Internal MedicineUniversity of MichiganAnn Arbor, MIUnited States; ^3^Center for Bioethics and Social Sciences in MedicineUniversity of MichiganAnn Arbor, MIUnited States; ^4^Risk Science CenterSchool of Public HealthUniversity of MichiganAnn Arbor, MIUnited States

**Keywords:** Risk, patient education as topic, patient-provider communication, decision aids, visual aids

## Abstract

**Background:**

The increasing use of computer-administered risk communications affords the potential to replace static risk graphics with animations that use motion cues to reinforce key risk messages. Research on the use of animated graphics, however, has yielded mixed findings, and little research exists to identify the specific animations that might improve risk knowledge and patients’ decision making.

**Objective:**

To test whether viewing animated forms of standard pictograph (icon array) risk graphics displaying risks of side effects would improve people’s ability to select the treatment with the lowest risk profile, as compared with viewing static images of the same risks.

**Methods:**

A total of 4198 members of a demographically diverse Internet panel read a scenario about two hypothetical treatments for thyroid cancer. Each treatment was described as equally effective but varied in side effects (with one option slightly better than the other). Participants were randomly assigned to receive all risk information in 1 of 10 pictograph formats in a quasi-factorial design. We compared a control condition of static grouped icons with a static scattered icon display and with 8 Flash-based animated versions that incorporated different combinations of (1) building the risk 1 icon at a time, (2) having scattered risk icons settle into a group, or (3) having scattered risk icons shuffle themselves (either automatically or by user control). We assessed participants’ ability to choose the better treatment (choice accuracy), their gist knowledge of side effects (knowledge accuracy), and their graph evaluation ratings, controlling for subjective numeracy and need for cognition.

**Results:**

When compared against static grouped-icon arrays, no animations significantly improved any outcomes, and most showed significant performance degradations. However, participants who received animations of grouped icons in which at-risk icons appeared 1 at a time performed as well on all outcomes as the static grouped-icon control group. Displays with scattered icons (static or animated) performed particularly poorly unless they included the settle animation that allowed users to view event icons grouped.

**Conclusions:**

Many combinations of animation, especially those with scattered icons that shuffle randomly, appear to inhibit knowledge accuracy in this context. Static pictographs that group risk icons, however, perform very well on measures of knowledge and choice accuracy. These findings parallel recent evidence in other data communication contexts that less can be more—that is, that simpler, more focused information presentation can result in improved understanding. Decision aid designers and health educators should proceed with caution when considering the use of animated risk graphics to compare two risks, given that evidence-based, static risk graphics appear optimal.

## Introduction

The most basic way to communicate risk to patients is to provide them with a risk number. All numerical formats are not equivalent, however, and considerable research has compared, for example, the pros and cons of using frequency formats instead of percentages [1-4] and the potential pitfalls of specific formats such as 1-in-X [5,6] and number needed to treat [7]. In addition, research on affective influences on risk perceptions make it clear that the same risk number can lead to very different feelings about that risk based on different circumstances, presentations, or contexts [8,9].

In an effort to make risk statistics more intuitive, both researchers and health educators have increasingly turned to visual displays of risk. While this literature is evolving, comparative studies have shown that icon-based displays (often called pictographs or icon arrays) appear to have significant advantages over other displays such as bar graphs and pie charts [10-14]. In particular, displays that visually represent the part-whole relationships (ie, the number of risk events in comparison with the entire at-risk population) appear to be better understood [14,15] and may be of particular help to people with lower numeracy skills [16].

### Animated Risk Displays

While most numerical and visual formats for communicating risk are easily implementable in traditional paper-and-pencil materials, the last decade has seen an enormous increase in the use of electronic applications designed to communicate risk to patients. Today, we regularly see computers in clinical consultation rooms, patients can go to innumerable websites that will estimate their health risks, and a growing suite of mobile device applications are available that purport to support healthy living in various ways.

Such technologies open the door to using multimedia techniques such as animation for communicating risk. Animation is commonly used in online applications to call users’ attention to a particular area of the screen [17] or portion of the content [18]. This type of signaling may help people make sense of verbal information [19], may help them better learn how a complex system such as a turbofan jet engine works [20], and may help somewhat in the acquisition of complex cognitive skills such as doing experimental research or designing a house [21].

### Motion Cues

Animation of risk graphics could be particularly useful because it allows motion cues to draw attention to specific elements of the visual display. For example, instead of simply showing the proportion of area or icons affected by a risk, animation could be used to sequentially draw the viewer’s eye to each new risk event, thereby adding a time cue (how quickly the set of events occurs) to reinforce the smallness or largeness of the risk. Such cues may be particularly useful when comparing multiple risks.

### Transformations

When people are at risk for a health outcome, they will either experience the outcome or not. The randomness of this occurrence or nonoccurrence is one of the conceptual challenges of risk communication. Past research has noted that icon array displays that scatter event icons randomly convey this sense of randomness but make it difficult to grasp exactly how large the risk is [3,22], whereas displays that group event icons are easy to count (allowing for faster interpretation) [11]. Animation could be used to transform one arrangement into another (for example, a scattered display into a grouped display, or one type of scatter into another), which might enable viewers to have the best of both worlds.

### Potential Concerns

While animated displays may have potential advantages, there are also several reasons for caution and further research regarding their use in risk communication applications. Many types of animation exist, each of which may reinforce different types of gist messages. In the absence of research clarifying what types of animation are taken to imply different types of conceptual understanding, use of animated graphics has the potential to cause unintended negative effects. Reviews of other types of animated graphics have found decidedly mixed results [23], providing reason to question whether animated graphics will support improved understanding over static graphs. It may be the case that their utility depends on user characteristics. For example, animated graphs have previously been demonstrated to help people who had performed well on a short mathematics test administered prior to an experiment that tested ability to transform graphs of mathematical functions, while hindering those who had performed more poorly on the mathematics test [24]. The benefits or drawbacks of adding animation cues may also depend on the complexity of the visual stimulus, since human factors research has shown that excessively complicated displays can reduce people’s ability to attend to particular cues [18].

### Existing Research

Very little work has been done to assess whether animation of risk graphics can improve people’s understanding of their health risk, or which types of animation might improve or inhibit accuracy of risk knowledge and risk perceptions. One notable exception is the study of Han et al, which showed that a dynamic scattered display increased subjective uncertainty about a risk, making participants less certain about their interpretations of risk information [25]. Other researchers have examined using interactive risk displays to engage people in learning about risk [26-28], but these studies have focused on the effect of different types of participant tasks (eg, manually graphing a provided risk number or playing within a game-like environment) rather than the visual cues potentially provided by animation.

To begin to explore whether viewing specific types of motion cues and visual transformations in animated risk graphics could improve people’s knowledge accuracy, decision making or choice accuracy, and graph evaluation ratings as compared with viewing static images, we conducted a randomized survey experiment comparing various animated and static displays in the context of a hypothetical medical treatment scenario. Our primary research question was whether animated displays with different types of motion would increase or decrease participants’ ability to identify which of two treatment options had lower side effect risks (choice accuracy). A secondary research question involved determining, via graph evaluation ratings, whether people like or dislike these types of animation for receiving medical risk information.

## Methods

### Recruitment

We selected a stratified random sample of US adults age 21 years and older from a panel of Internet users administered by Survey Sampling International (Shelton, CT, USA), which recruits panel members through a variety of opt-in methods. To ensure demographic diversity (though not necessarily representativeness) and offset large expected variations in response rates, we drew distinct subsamples by both age and race (thereby roughly approximating the distributions of these characteristics in the US population), and dynamically adjusted the number of email invitations in each demographic subsample until all quotas were achieved. Selected panel members received an email invitation with a personalized link to complete the online survey with one reminder email for nonresponders. Survey Sampling International tracked participation via unique identification numbers to prevent duplicate uses of the same link to participate. We recruited for a 3-week period in fall 2010. On completion, participants were entered into both an instant-win contest and a monthly drawing administered by Survey Sampling International for modest prizes.

### Design of the Study

Respondents read a revised version of a short vignette previously used in a study of interactive graphics [28] in which they imagined being given a diagnosis of thyroid cancer and discussing two types of hypothetical external beam radiation treatments with their doctor. The two treatments, called focal beam therapy and crossed beam therapy, were each briefly described and then presented as being equally effective in treating the patient’s type of thyroid cancer. Each therapy also had the same risk (11%) of causing one side effect: fatigue. However, the treatments were described as differentially likely to cause a second side effect, mouth and throat problems: one therapy caused mouth and throat problems in 16% of patients, whereas the other therapy caused mouth and throat problems in only 14% of patients. We randomly assigned which therapy had the higher risk to prevent our scenario descriptions from biasing our results. The scenario referred to a less common disease (thyroid cancer, instead of breast or prostate cancer) and included hypothetical types of radiation beam therapy in order to minimize respondent preconceptions about treatment options or their associated risks.

Our primary research question was to determine whether different graphical formats would increase respondents’ ability to recognize which treatment option was less risky (choice accuracy). To do so, we implemented a quasi-factorial design to experimentally vary the type of risk graphic used to present each of the two side effects in a side-by-side presentation. Participants were randomly assigned to 1 of 10 experimental conditions summarized in Table 1.

**Table 1:** Experimental conditions.

Version		Animated?	Initial arrangement	Animation type		
				Built?	Settle into group?	Shuffle?
V1	Static grouped	No	Grouped	NA^a^	NA	NA
V2	Static scattered	No	Scattered	NA	NA	NA
V3	Scatter, settles	Yes	Scattered	No	Yes	No
V4	Grouped, built	Yes	Grouped	Yes	NA	No
V5	Scatter, built	Yes	Scattered	Yes	No	No
V6	Scatter, built, settles	Yes	Scattered	Yes	Yes	No
V7	Scatter, auto shuffles	Yes	Scattered	No	No	Automatic
V8	Scatter, auto shuffles, settles	Yes	Scattered	No	Yes	Automatic
V9	Scatter, user shuffles	Yes	Scattered	No	No	User controlled
V10	Scatter, user shuffles, settles	Yes	Scattered	No	Yes	User controlled

^a ^Not applicable.

**Figure 1:**
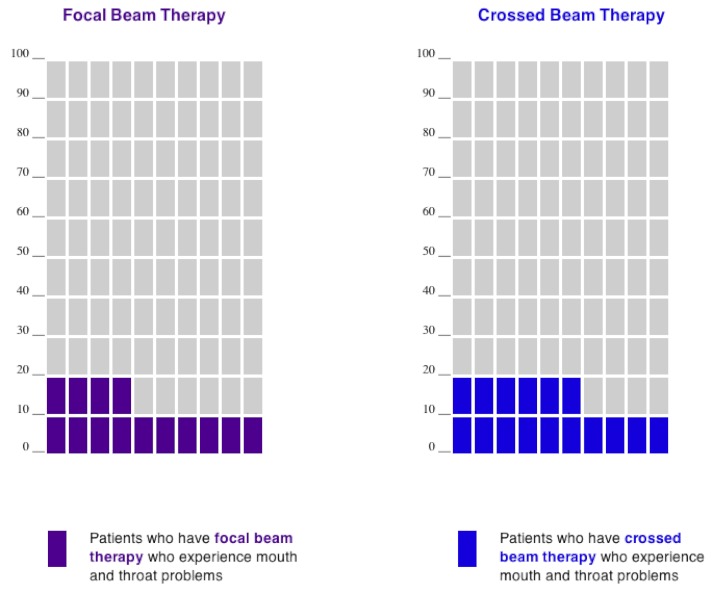
Icon arrays (version V1: static grouped) displaying the risk of mouth and throat problems for both hypothetical treatments.

Approximately 20% of participants in total were assigned to 1 of 2 static display conditions. Participants in the baseline condition (V1: static grouped) saw side-by-side static icon arrays (a 10 × 10 matrix of blocks) in which all of the colored blocks used to represent event occurrence (ie, experience of fatigue, or mouth or throat problems) were grouped at the bottom of the display (Figure 1). A second group of participants (V2: static scattered) viewed a scattered static display in which the event icons were randomly distributed within the matrix to help convey the underlying random distribution of events. Previous research has suggested that this type of scattered display does, in fact, help convey randomness, but at the expense of a sense of the magnitude of the risk [3,11,22,25]. We included this design factor to explore whether we might achieve the best of both worlds by displaying randomness without sacrificing a sense of quantity.

The remaining 80% of participants viewed 1 of 8 animated displays that included 1 or more animations based on the different types of potentially useful motion cues discussed above. Some groups (V4, V5, and V6) viewed built displays that were either grouped or scattered according to the above description. In these versions, participants initially viewed an empty array (ie, all icons were gray) but then saw colored icons representing risk events appear sequentially (1 every 450 milliseconds) in each of the 2 graphs until the final level of risk was reached. Note that due to different levels of risk between the two treatment options, this animation meant that, for the display of mouth and throat problem risks, colored blocks finished appearing in 1 of the 2 arrays before the other one, creating a motion cue to reinforce which treatment had a larger risk.

Participants in the scattered graphs conditions (built or not) were further subdivided based on whether they saw 2 other animations. First, some participants in scattered conditions saw the scattered risk elements remain still (V2 and V5); others (V7 and V8) saw these colored blocks shuffle (redisplay themselves repeatedly in new randomly generated positions) in a manner similar to the dynamic random visual used by Han et al [25] to promote subjective uncertainty; and others (V9 and V10) were required to press a button that caused the blocks to shuffle a few times before they could proceed. We included this last condition to test whether having user control of the random scattering process would affect participants’ perceptions of the risk. Second, while some participants saw only the pictographs with the risk scattered (V2, V5, V7, and V9), others initially saw a scattered display that then showed the colored units settling down toward the bottom of the array and then arranging themselves into the same grouping seen by the grouped conditions. In other words, we used animation in these settle conditions (V3, V6, V8, and V10) to enable participants to observe both an initial scattered visual (which may promote understanding of randomness) and an ending grouped visual (to facilitate assessment of risk magnitude).

All survey versions were pretested by study team members for functionality and to estimate time to complete prior to survey launch. We also randomly varied which treatment was shown on the left as well as what color was used to refer to each treatment to prevent order effects. Example movies of V4 (grouped, built), V6 (scatter, built, settles), and V9 (scatter, user shuffles), which collectively demonstrate all of the animation types, are available as multimedia appendices (see Multimedia Appendix 1, Multimedia Appendix 2, and Multimedia Appendix 3).

On entering our survey, participants were given an introduction page that explained the purpose of the study, the anonymous nature of the research, and the expected time to take the survey. The survey consisted of 57 total questions over 20 webpages (between 0 and 9 questions per page). Participants also completed between 3 and 8 webpages of survey materials for unrelated studies (cross-randomized across all 10 arms of this study) after completing the primary and secondary measures for this study but before completing individual difference measures (eg, numeracy) and demographics. This design received institutional review board exempt status approval as anonymous survey research.

### Measures and Covariates

Our primary outcome measure was the preferred treatment choice (focal beam or crossed beam). We also asked respondents two gist knowledge questions in which they were to indicate which therapy had a higher risk of fatigue (both equal), or mouth or throat problems (which varied based on randomization).

We asked 3 graph evaluation items to evaluate user preferences about the different risk graphics. These questions asked respondents to use a 10-point Likert-type scale to rate how well the graphs described the risk of different side effects, how helpful the graphs were, and whether the respondent would like to see risk information in this type of graph. Our a priori intention was to combine these ratings into a 3-item scale.

Because ample evidence exists that even highly educated adults can have poor numeracy skills (ie, facility and comfort with quantitative health information such as risk statistics) [29-31], all study participants also completed the Subjective Numeracy Scale (SNS) [32], a validated measure of quantitative ability and preference for receiving information in numerical form. The SNS has previously been shown to correlate with the ability to recall and comprehend both textual and graphical risk communications [33,34]. A participant’s SNS score is calculated as his or her mean rating across the 8 SNS questions and ranges from 1 (least numerate) to 6 (most numerate).

We also assessed participants’ need for cognition using a shortened version (7 of 18 questions) of the Need for Cognition Scale [35] due to concerns about survey duration. Responses were averaged to create a single scale.

### Statistical Analyses

Since our main goal for this study was to explore the effect of different types of animation on treatment choices, primary analyses focused on the percentage of accurate choices (ie, percentage choosing the treatment with the lowest risk profile), knowledge accuracy, and graph evaluation ratings. To calculate the significance of the observed variations, we used logistic regression models (linear regressions for graph ratings) that included graph version (with V1: static grouped as reference), as well as SNS score and Need for Cognition Scale score as covariates. We also analyzed subsets for the effect of numeracy by splitting at the median, grouping participants into lower-numeracy and higher-numeracy subgroups, and rerunning the logistic regression analyses for each numeracy subgroup (while still controlling for need for cognition). All analyses were performed using Stata version 11 [36], and all tests of significance were 2-sided and used alpha = .05.

## Results

### Sample Description

In total, 6240 people age 21 years and older reached the survey website and viewed the first content page. Of these, 38 reported having a diagnosis of thyroid cancer and were excluded as having preexisting knowledge of related treatment options, leaving 6202 possible participants.

Overall, 4198 (67.7%) of participants completed the entire survey (range across the 10 survey versions: 64%–71%), including questions on demographics, which came toward the end of the survey instrument. We restricted our analyses to this subsample. Characteristics of those participants who answered each demographic question are reported in Table 2. We observed a wide range of educational achievement, with 1540 participants (36.7%) having a bachelor’s or higher college degree but also 809 (19.3%) having completed high school or less education. The SNS numeracy measure showed high reliability (Cronbach's alpha = .86), as did the shortened need for cognition measure (Cronbach alpha = .83). Mean SNS score was 4.30, with substantial variation (range 1.5–6.0; SD 1.03). Because questions about participants’ demographics came at the end of the survey, we do not know whether the demographics of those who dropped out differed from those who completed the survey.

**Table 2:** Participant demographic characteristics (n = 4198)^a^.

Characteristic	Category	Frequency (%)	Mean (SD)
Age (years)	21–29	698 (16.7%)	49.1 (16.1)
	30–39	663 (15.8%)	
	40–49	588 (14.0%)	
	50–59	848 (20.2%)	
	60–69	1035 (24.7%)	
	70+	359 (8.6%)	
Gender	Male	1936 (46.2%	
	Female	2255 (53.8%)	
Ethnicity	Hispanic (any race)	485 (11.7%)	
Race^b^	White	3267 (78.0%)	
	African American	592 (14.1%)	
	All other	384 (9.2%)	
Education	< High school	86 (2.1%)	
	High school only	723 (17.3%)	
	Some college/trade	1832 (43.8%)	
	Bachelor’s degree	1017 (24.3%)	
	Master’s/doctorate	523 (12.5%)	
Subjective Numeracy Scale score	1.00–1.99	102 (2.4%)	4.30 (1.03)
	2.00–2.99	337 (8.1%)	
	3.00–3.99	970 (23.3%)	
	4.00–4.99	1491 (35.8%)	
	5.00–5.99	1179 (28.3%)	
	6.00	91 (2.2%)	

^a ^Reports results only for those respondents who completed each question or measure.

^b ^Respondents could mark more than one race.

### Treatment Choice Accuracy

Table 3 reports the percentage of participants who correctly chose the dominant treatment option (ie, the therapy with the lower risk of mouth and throat problems), stratified by numeracy level. Lower-numeracy participants selected the best treatment option about 75% of the time, and varying the graphic used to display the side effect risks did not result in any significant differences in treatment choices based on the logistic regression analysis. There was much more variation, however, among higher-numeracy participants. While participants in the baseline V1 group (static grouped) made the correct treatment choice about 85% of the time, participants in most of the other experimental conditions were less likely to pick the best treatment choice. In the logistic regression analysis, we observed significantly different selection rates for participants in all of the scattered but not settled groups (V2, V5, V7, and V9) as well as V10 (scatter, user shuffles, settles). Most of the remaining experimental groups also were less likely than the V1 control group to choose the best treatment, although the differences were not statistically significant. The only group that was more likely (albeit not statistically significant) than the static grouped condition to choose optimally was V4 (built, grouped).

**Table 3:** Percentage choosing best treatment option, by graph version and respondent numeracy.

Version		Lower numeracy			Higher numeracy		
		%	OR^a^ (95% CI^b^)	*P* value	%	OR (95% CI)	*P* value
V1	Static grouped	74.3	Reference	–	84.8	Reference	–
V2	Static scattered	72.2	0.90 (0.59–1.36)	.62	76.6	0.58 (0.34–1.00)	.05
V3	Scatter, settles	75.5	1.04 (0.68–1.59)	.85	80.0	0.72 (0.42–1.24)	.23
V4	Grouped, built	75.2	1.02 (0.67–1.55)	.93	86.3	1.14 (0.64–2.04)	.66
V5	Scatter, built	68.3	0.73 (0.48–1.11)	.14	74.4	0.53 (0.31–0.90)	.02
V6	Scatter, built, settles	77.3	1.14 (0.74–1.77)	.55	81.0	0.77 (0.44–1.34)	.35
V7	Scatter, auto shuffles	72.3	0.87 (0.56–1.34)	.52	74.2	0.52 (0.31–0.89)	.02
V8	Scatter, auto shuffles, settles	76.4	1.08 (0.70–1.67)	.72	82.9	0.87 (0.50–1.50)	.61
V9	Scatter, user shuffles	72.9	0.92 (0.60–1.40)	.69	67.6	0.37 (0.22–0.63)	<.001
V10	Scatter, user shuffles, settles	73.3	0.94 (0.62–1.43)	.77	75.8	0.56 (0.33–0.96)	.03

^a ^Odds ratio reported from logistic regression model controlling for need for cognition.

^b ^Confidence interval.

### Gist Knowledge Accuracy

Table 4 reports the percentage of participants (by graph version and numeracy level) who accurately identified that both treatments had equal risks of fatigue. Here, the pattern of results is quite similar for both lower- and higher-numeracy respondents. In both numeracy groups, the baseline group (V1) that saw static grouped graphs had either the highest or next-highest level of knowledge. Knowledge was similar to the baseline in V4 (grouped, built) for lower-numeracy participants, and in V8 (scatter, shuffles, settles) for all participants. Participants in the remaining groups all showed lower knowledge rates, with statistically significant differences observed in the logistic regression analyses for V2, V5 (higher numeracy only), V7 (lower numeracy only), V9 and V10 (higher numeracy only).

The pattern of results was quite similar for accurately identifying the treatment with the higher rates of mouth and throat problems (Table 5), with lower knowledge rates observed for versions V2, V5, V7, and V9 in particular. However, people who saw the V4 (grouped, built) risk graphic had somewhat higher knowledge than those who saw the baseline V1 (static grouped) version, although this difference was not significant for either lower- or higher-numeracy participants.

**Table 4:** Percentage correctly identifying that both treatments had an equal risk of fatigue, by graph version and respondent numeracy.

Version		Lower numeracy			Higher numeracy		
		%	OR^a^ (95% CI^b^)	*P* value	%	OR (95% CI)	*P* value
V1	Static grouped	78.8	Reference	–	86.3	Reference	–
V2	Static scattered	68.8	0.59 (0.38–0.91)	.02	73.3	0.44 (0.26–0.75)	.003
V3	Scatter, settles	71.6	0.67 (0.43–1.03)	.07	81.2	0.69 (0.39–1.22)	.21
V4	Grouped, built	79.3	1.01 (0.64–1.60)	.97	82.1	0.74 (0.42–1.32)	.31
V5	Scatter, built	74.0	0.75 (0.48–1.18)	.21	75.0	0.49 (0.28–0.84)	.01
V6	Scatter, built, settles	75.3	0.80 (0.51–1.26)	.34	83.1	0.79 (0.44–1.43)	.44
V7	Scatter, auto shuffles	70.9	0.64 (0.40–1.00)	.05	84.1	0.85 (0.47–1.54)	.60
V8	Scatter, auto shuffles, settles	78.2	0.94 (0.59–1.49)	.79	86.2	0.99 (0.55–1.79)	.98
V9	Scatter, user shuffles	66.1	0.52 (0.34–0.80)	.003	70.2	0.38 (0.22–0.64)	<.001
V10	Scatter, user shuffles, settles	75.3	0.81 (0.52–1.27)	.36	77.7	0.56 (0.32–0.98)	.04

^a ^Odds ratio reported from logistic regression model controlling for need for cognition.

^b ^Confidence interval.

**Table 5:** Percentage correctly identifying the treatment with the higher risk of mouth and throat problems, by graph version and respondent numeracy.

Version		Lower numeracy			Higher numeracy		
		%	OR^a^ (95% CI^b^)	*P* value	%	OR (95% CI)	*P* value
V1	Static grouped	46.9	Reference	–	65.1	Reference	–
V2	Static scattered	44.9	0.92 (0.64–1.34)	.68	55.7	0.68 (0.44–1.04)	.07
V3	Scatter, settles	45.5	0.93 (0.64–1.33)	.68	62.3	0.89 (0.58–1.36)	.59
V4	Grouped, built	54.9	1.35 (0.94–1.95)	.11	69.8	1.25 (0.81–1.95)	.31
V5	Scatter, built	42.4	0.81 (0.55–1.19)	.29	55.0	0.67 (0.43–1.02)	.06
V6	Scatter, built, settles	50.9	1.15 (0.79–1.67)	.46	62.9	0.92 (0.59–1.42)	.70
V7	Scatter, auto shuffles	39.4	0.71 (0.48–1.05)	.09	52.6	0.60 (0.39–0.93	.02
V8	Scatter, auto shuffles, settles	53.0	1.24 (0.85–1.80	.26	64.9	0.99 (0.65–1.52)	.96
V9	Scatter, user shuffles	34.6	0.59 (0.41–0.87)	.007	48.6	0.51 (0.33–0.78)	.002
V10	Scatter, user shuffles, settles	42.9	0.84 (0.58–1.22)	.36	54.8	0.76 (0.50–1.16)	.21

^a ^Odds ratio reported from logistic regression model controlling for need for cognition.

^b ^Confidence interval.

### Graph Evaluation Ratings

As planned, we combined our 3 graph evaluation rating questions into a 3-item scale based on the average rating, which had high reliability (Cronbach’s alpha = .93). Table 6 reports the mean graph evaluation ratings for each graph type. Here, a clear pattern emerges: consistent with the previous results for knowledge accuracy and treatment choice accuracy, participants in 2 conditions, V1 (static grouped) and V4 (grouped, built), reported the highest evaluation ratings for both lower- and higher-numeracy participants. Participants in the V3 (scatter, settles) and V6 (scatter, built, settles) groups had slightly lower ratings, though the differences were not statistically significant. The remaining 6 graph types received significantly lower graph evaluation ratings in the linear regression models, with all differences highly significant (all *P *< .001) versus the baseline static grouped condition.

**Table 6:** Graph evaluation ratings^a^, by graph version and respondent numeracy.

Version		Lower numeracy			Higher numeracy		
		Mean	Coefficient^b^ (95% CI)^c^	*P* value	Mean	Coefficient (95% CI)	*P* value
V1	Static grouped	5.99	Reference	–	7.24	Reference	–
V2	Static scattered	4.68	–1.30 (–1.75 to –0.86)	<.001	5.07	–2.17 (–2.68 to –1.65)	<.001
V3	Scatter, settles	5.72	–0.29 (–0.73 to 0.15)	.19	6.72	–0.51 (–1.03 to 0.00)	.05
V4	Grouped, built	6.31	0.30 (–0.14 to 0.75)	.19	6.98	–0.25 (–0.77 to 0.26)	.33
V5	Scatter, built	4.89	–1.12 (–1.59 to –0.66)	<.001	5.60	–1.64 (–2.15 to –1.12)	<.001
V6	Scatter, built, settles	5.90	–0.12 (–0.57 to 0.34)	.62	6.81	–0.43 (–0.95 to 0.09)	.11
V7	Scatter, auto shuffles	4.02	–2.01 (2.48 to –1.55)	<.001	4.60	–2.64 (–3.16 to –2.12)	<.001
V8	Scatter, auto shuffles, settles	5.27	–0.75 (–1.20 to –0.29)	<.001	6.08	–1.16 (–1.66 to –0.65)	<.001
V9	Scatter, user shuffles	4.09	–1.91 (–2.36 to –1.46)	<.001	4.77	–2.47 (–2.98 to –1.95)	<.001
V10	Scatter, user shuffles, settles	4.88	–1.12 (–1.57 to –0.67)	<.001	5.34	–1.90 (–2.41 to –1.39)	<.001

^a ^Ratings are the average of 3 questions, each reported on a 0–9 scale.

^b ^Coefficient from linear regression model controlling for need for cognition.

^c ^Confidence interval.

## Discussion

### Principal Results

In this study, we evaluated 8 different animated icon array risk graphics that incorporated different combinations of 3 basic animations: building risk 1 unit at a time, settling scattered risk into a grouping to ease assessment of magnitude, and shuffling scattered risk to reinforce randomness. When compared against the type of static, grouped icon pictographs that have been previously shown to support high levels of risk knowledge [10], the animated graphics consistently fell short. No animated display resulted in significantly improved knowledge or evaluation ratings versus the static grouped control display, and significant deficits were observed for most of the animated versions. Only the building animation that presented the colored icons representing risk events 1 at a time (eg, V4: grouped, built) showed even the slightest promise of improving understanding, and this was not consistent across outcome measures.

Consistent with some prior research [22], scattered risk displays generally resulted in poorer knowledge and graph evaluation ratings. Shuffling the event icons often made things worse and dramatically lowered evaluation ratings. Adding an animation to allow a scattered risk to settle into a grouping did help, though such animations did not convey any advantages over displays that started in a grouped orientation to begin with. However, a parallel study from our research group (personal communication with HO Witteman, et al, December 8, 2011) suggested that animated displays of scattered icons that include both the building and settling animations may increase sensitivity to differences in risk magnitude. In addition, Han et al found that a dynamic scattered icon array resulted in increased subjective uncertainty about cancer risks [25]. If so, a scatter-plus-settle animation may have practical value even if it does not confer intrinsic improvements in risk knowledge or preference.

### Limitations

Our study has several key limitations. First, we recruited participants from an online survey panel and gave them a hypothetical medical treatment scenario. As a result, participants may well have been less motivated to learn about the risk levels and more easily distracted by the animations. This account is consistent with our findings that increased complexity of animations (eg, shuffling) particularly decreased participant knowledge accuracy. It is certainly possible that patients facing actual medical treatment decisions would be better focused on the risk knowledge and, as a result, have smaller deficits or perhaps improvements in understanding over static graphs. We note, however, we found our strongest variations not in knowledge, but in our participants’ graph evaluation ratings. Additionally, it is possible that patients facing actual medical treatment decisions would be more susceptible to distraction due to the complexity of animations because of increased cognitive burden or stress brought about by their illness. Although it is plausible that more complicated, “cooler” animations might yield higher evaluation ratings, especially among participants who were only taking a survey and not making real medical decisions, in fact we observed the opposite pattern: the most complex graphics were least preferred by our study participants.

Second, the task used in our experiment required comparing two risks. Animated graphs were presented side-by-side, making it possible that it was the dual presentation of animation, rather than the animation per se, that hampered the communicative effectiveness of the graphics. We selected a comparison task because many risk evaluation and decision processes require balancing competing risks and benefits, making this a plausible application for such graphics. It may be that, although the animation was harmful in this context, it might still hold value in the context of a single risk, or when presenting 1 risk at a time.

Third, our analyses focused here on differences between higher-numeracy and lower-numeracy participants. However, recent research has shown that interpretation of risk graphics is also mediated by graphical literacy skills, which are only moderately correlated with numeracy [37,38]. It is possible that some of the effects we attribute to numeracy are in fact graphical literacy effects. While we did not collect graphical literacy measures here (because these data were collected prior to publication of the scale), we intend to measure both numeracy and graphical literacy in follow-up research.

### Comparison With Prior Work

Our study is placed in the context of previous work, most notably Tversky et al’s 2002 extensive review of animated graphics [23]. Using their delineation, the task of specifying a dominant option in our study is made more challenging by the fact that health risks are not inherently visuospatial concepts. In their review, the authors noted mixed effects of animated graphics, and suggested that fair comparisons between animated graphics and static graphics require information content that can be adequately conveyed in the static form. This was the case in our context, and this may explain the lack of benefit demonstrated, similar to previous studies where static graphics were able to effectively convey information (eg, [39]). Tversky et al [23] also reviewed other experimental studies, based on which they argued, reasonably, that the benefits of some animated graphics may be attributable to the additional information content that could be conveyed via the animated movement. Therefore, potential benefits of animation may exist, but are concentrated in contexts in which static graphics cannot communicate all the information of the animated versions. The near equivalence of some of our animated versions in performance measures suggests that further research is needed to investigate their potential for conveying additional information that is difficult to convey in static graphics alone, for example, the random nature of events in health risks.

It is also important to note that our task in this study involved the comparison of two risks. This is a common issue in assessing health risks and making decisions accordingly. However, this may have introduced problems by dividing participants’ attention between two areas of the screen, both of which were moving simultaneously. This may reflect the fact that people find focusing on competing animations difficult [17].

Previous work suggests an interaction between domain knowledge and the effects of animation. For example, animation helped more advanced students learn to transform graphs of simple mathematical functions into more complex functions but hindered novice students [24]. Our study, on the other hand, suggests that the performance of people with lower numeracy, who might be expected to have more trouble with animation, did not differ across conditions, whereas those with higher numeracy showed a decline in performance with the addition of animation (while still maintaining higher overall rates of accuracy and knowledge). We speculate that this effect depends on whether the animation builds on prior knowledge (as may, for example, be the case with animated displays of physics problems) or distracts from people’s ability to perform required tasks. Recent work has shown that more numerate people tend to count icons in displays such as ours and derive their sense of risk magnitude from that process [40]. In our experiments, the attention-grabbing nature of the animation may have prevented higher-numeracy participants from applying this learning strategy, thereby degrading their performance.

It is also worth clarifying the distinction between the animated risk graphics tested here and interactive graphing tasks in which the message recipient has to alter the visual display to show or uncover risk information. Researchers have tested the impact of having people adjust bar graphs [26] or pictographs [28] to display provided risk statistics. However, these studies have had decidedly mixed effects, with the pictograph study finding that the interactive task significantly decreased people’s ability to identify a dominant treatment option [28]. Another recent study used an exploratory task in which participants clicked in a matrix until they uncovered a risk event. This task elicited more emotional responses than static graphics, increasing qualitative statements of concern about large risks or relief about small ones. However, a subsequent experimental study found no overall effect of interactive versus static graphic type on risk estimates or risk feelings, though it did reduce disparities attributable to differences in numeracy [27]. Such mixed findings are mirrored in research on other forms of interactivity in health education such as video games [41] and immersive 3-dimensional environments [42].

There are also considerable parallels between our findings regarding the potentially distracting effects of animation and recent evidence in other data communication contexts that less can be more—that is, that simpler, more focused information presentations can result in improved understanding. For example, people are better able to identify preferred hospitals out of a set when tabular presentations of data excluded decision-irrelevant information [43]. Similarly, understanding of cancer recurrence risks and decisions about adjuvant therapies can be improved by removing information about irrelevant options [12], excluding redundant mortality statistics [44], and presenting relevant information one piece at a time [34]. Both these studies and our present investigation serve as reminders that people’s ability to process multiple things at once is quite limited, and thus risk communications need to ensure that the user’s attention is drawn narrowly and specifically to the most important piece of data or visual cue. In terms of animation, both our study and Tversky and colleagues’ [23] review imply that static graphics may be preferable to animated versions as long as the static displays fully present the most decision-relevant information.

### Conclusions

If the goal of a health risk communication is to ensure that patients understand the magnitude of risk and are able to make appropriate comparisons between two risks, our work suggests that the use of animation to provide motion cues in computer-administered risk graphics is fraught with peril. We tested 8 combinations of 3 core animations that we believed might support better understanding or satisfaction, but our results showed that these animations were at best unhelpful and often significantly detrimental. Static pictographs that grouped event icons at the bottom of the array consistently resulted in optimal treatment choices, higher knowledge accuracy, and better graph evaluation ratings. This finding adds to the growing literature supporting their use as best practice in many patient education contexts.

Computer-based communications are likely to be the mode of choice for many future efforts to educate patients about health risks that require preventive action and medical treatment decisions. Such technologies offer many new types of visual (and auditory) cues that could be used to reinforce risk information, and there are often pressures to use the latest “bells and whistles” in such applications. Our research, however, sounds a cautionary note.

Ultimately, effective patient decision making requires specific types of understanding. At a minimum, patients need to realize that a risk could occur and be able to identify which actions are more or less likely to lead to preferred outcomes. While we remain hopeful that certain types of animation might be useful in specific risk communication contexts (eg, by using building animations to show accumulation of risk over time), our present efforts did not support quality decision making. More research is clearly needed to evaluate different types of motion cues and to identify which animations lead to better results versus the features of those that do not. In the meantime, we reiterate the finding from our previous work in interactive graphics [28]: decision aid designers should proceed with caution when considering the use of flashy risk graphics.

## Multimedia Appendix 1

MP4 movie showing the V4 (Grouped, Built) animation of the risk of mouth and throat problems.

## Multimedia Appendix 2

MP4 movie showing the V6 (Scatter, Built, Settles) animation of the risk of mouth and throat problems.

## Multimedia Appendix 3

MP4 movie showing the V9 (Scatter, User Shuffles) animation of the risk of mouth and throat problems.

## Abbreviations

SNS: Subjective Numeracy Scale
